# Supplementary material for: The C. elegans immune switch proteins PALS-25 and PALS-22 localize to mitochondria and regulate fragmentation
Source: bioRxiv. 2025 Oct 15:2025.10.13.682198. Preprint. [Version 1] doi: 10.1101/2025.10.13.682198 (PMC12633051; doi:10.1101/2025.10.13.682198)
Supplement: Supplement 1 [file NIHPP2025.10.13.682198v1-supplement-1.pdf]

bioRxiv preprint doi: https://doi.org/10.1101/2025.10.13.682198; this version posted October 15, 2025. The copyright holder for this preprint (which was not certified by peer review) is the author/funder, who has granted bioRxiv a license to display the preprint in perpetuity. It is made available under aCC-BY 4.0 International license.

Figure S1. PALS-22 and PALS-25 expression vectors used in this study, and intestinal localization of PALS-22::GFP. A-B) Simplified vector maps of the TransgeneOme fosmid constructs used to express PALS-22::GFP and PALS-25::GFP. **A)** *jyEx193[pals-22p::pals-22::GFP::3xFLAG::sl2::pals-25, unc-119(+)]* expresses PALS-22 C-terminally tagged with GFP::3xFLAG and wild-type PALS-25 from the same fosmid using the endogenous *pals-22/25* regulatory elements. **B)** *jyEx237[pals-22p::pals-22::sl2::pals-25::GFP::3xFLAG, unc-119(+)]* expresses PALS-25 C-terminally tagged with GFP::3xFLAG and wild-type PALS-22 from the same fosmid using the endogenous *pals-22/25* regulatory elements. **C)** The *pals-22* and *pals-25* genomic locus. *pals-22* (orange exons) and *pals-25* (blue exons) are in an operon. **D)** PALS-22::GFP colocalizes with the mitochondria marker MitoTracker Red in the intestine. The first ring of intestinal cells of an adult animal is shown. White arrows denote PALS-22::GFP localized to mitochondria, white triangles denote autofluorescent gut granules. Scale bar = 10 μm. **E**) Vector map of pET787 used to co-express fluorescently tagged GFP::PALS-22 and mScarlet::PALS-25 using a smaller DNA construct lacking endogenous introns but maintaining the endogenous promoter and SL2 sequence for *pals-22/25.*

Figure S2. PALS-22::GFP and PALS-25::GFP are functional for regulating the IPR. A) RT-qPCR of select IPR-induced genes (*pals-5*, *F26F2.1*, and *skr-5)* in *pals-22 pals-25(jy80)* double mutants expressing *jyEx193* following either control RNAi or *pals-22* RNAi. IPR gene expression is induced following *pals-22* RNAi, indicating that PALS-22::GFP expressed from *jyEx193* is functional for the repression of PALS-25. **B)** RT-qPCR of select IPR-induced genes (*pals-5*, *F26F2.1*, and *skr-5)* in *pals-22 pals-25(jy80)* double mutants expressing *jyEx237* following either control RNAi or *pals-22* RNAi. IPR gene expression is induced following *pals-22* RNAi, indicating that PALS-25::GFP expressed from *jyEx237* is functional for activating the IPR. For **A** and **B**, although *pals-22* and *pals-25* are co-transcribed in an operon, *pals-22* RNAi does not knock down *pals-25* transcript levels. * $p < 0.05$, *** $p < 0.001$, One-tailed t-test. **C)** RT-qPCR of select IPR-induced genes *pals-5*, *F26F2.1,* and control gene *nhr-23,* in *pals-22 pals-25(jy80)* double mutants in white, *pals-25(GOF)* in gray, *pals-22 pals-25(jy80)* double mutants expressing *jyEx193* in orange, and *pals-22 pals-25(jy80)* double mutants expressing *jyEx237* in blue. *pals-5* IPR gene expression is induced in *pals-25(GOF)* relative to *pals-22/25* double mutants but not in *pals-22/25* double mutants expressing *jyEx193* nor in *pals-22/25* double mutants expressing *jyEx237*, indicating that the IPR is not induced in basal conditions with these TransgeneOme fosmid constructs. *pals-25(GOF)* does not induce *F26F2.1,* which is known not to be induced in this

bioRxiv preprint doi: https://doi.org/10.1101/2025.10.13.682198; this version posted October 15, 2025. The copyright holder for this preprint (which was not certified by peer review) is the author/funder, who has granted bioRxiv a license to display the preprint in perpetuity. It is made available under aCC-BY 4.0 International license.

mutant (Gang et al. 2022). * $p < 0.05$, two-way ANOVA. For **A-C**, n = 4 independent experimental replicates, different symbol shapes represent the expression values for replicates performed on different days. The graphs indicate mean values and error bars represent standard deviations.

Figure S3. PALS-22 protein levels do not change upon knock-down of *pals-25,* but PALS-25 protein levels are reduced upon knock-down of *pals-22*. A) Representative images of PALS-22::GFP expressed in the epidermis of adult animals under control, *pals*-22, and *pals*-25 RNAi treatments. **B)** Left, Western blot analysis of PALS-22::GFP expression levels in total protein lysates following different RNAi treatments using custom antibodies against PALS-22 and commercially available antibodies against tubulin as a loading control. Right, Quantification of PALS-22::GFP levels in total protein lysates following the different RNAi treatments, normalized to tubulin. **C)** Left, Western blot analysis of PALS-25::GFP expression levels in total protein lysates following different RNAi treatments using custom antibodies against PALS-25 and commercially available antibodies against tubulin as a loading control. Right, Quantification of PALS-25::GFP levels in total protein lysates following the different RNAi treatments, normalized to tubulin. **D)** Representative images of PALS-25::GFP expressed in the epidermis of adult animals under control, *pals*-22, and *pals*-25 RNAi treatments. For **A** and **D**, scale bar = 20 μm. For **B** and **C**, * $p < 0.05$, *** $p < 0.001$, one-way ANOVA with Dunnett's multiple comparisons test compared to control RNAi-treated animals. n = 3 independent experimental replicates. The graphs indicate mean values and error bars represent standard deviations.

Figure S4. Images of mitochondria morphology upon loss of *pals-22* and/or *pals-25*. A) Representative images of *jyEx4796[col-19p::*mito-GFP*]* expression in the epidermis of wild-type adult animals treated with control or *pals-22* RNAi; *pals-22* RNAi alters mitochondrial morphology in the epidermis. **B)** Representative images of wild-type, *pals-22(jy3)*, and *pals-22 pals-25(jy80)* mutants stained with MitoTracker Red to visualize epidermal mitochondria morphology; *pals-22 pals-25(jy80)* mutants display grossly wild-type mitochondria morphology. Scale bar = 20 μm, ROI scale bar = 2.5 μm. **C)** mitoUPR upregulated genes constitutively induced by the *atfs-1(et15)* gain-of-function allele have significant overlap with the IPR. Hypergeometric test, RF = 2.2, $p < 4.036e\text{-}05$. **D)** Representative images of *mgIs48[ges-1p::mito-GFP]* animals treated with control or *drp-1* RNAi. *drp-1* RNAi alters mitochondrial morphology in the intestine. **E)** Representative images of *pals-22(jy3); mgIs48[ges-1p::mito-GFP]* animals treated with control, *drp-1,* or *pink-1* RNAi. *drp-1* and *pink-1* RNAi do not rescue mitochondrial morphology defects observed in *pals-22* mutant animals. For **A, D,** and **E**, scale bar = 10 μm, ROI scale bar = 2.5 μm.

bioRxiv preprint doi: https://doi.org/10.1101/2025.10.13.682198; this version posted October 15, 2025. The copyright holder for this preprint (which was not certified by peer review) is the author/funder, who has granted bioRxiv a license to display the preprint in perpetuity. It is made available under aCC-BY 4.0 International license.

Figure S5. IPR reporter expression is induced in multiple tissues following 3 hours of auxin-mediated depletion of PALS-22. IPR reporter expression in transgenic animals expressing ubiquitous TIR1, endogenous *pals-22* tagged with AID, and the *pals-5p::*GFP, *myo-2p::*mCherry IPR reporter treated with vehicle control (top two rows) or auxin (bottom two rows) for 3 h starting at the L4 life stage. Top images for each treatment are an overlay of DIC, GFP, and mCherry fluorescence channels. Bottom images are GFP alone and indicate the induction of the *pals-5p::*GFP IPR reporter. IPR expression is visible after 3 h auxin treatment in multiple tissues; left to right = the anterior intestine, the epidermis (seam cells denoted by the white triangle; other epidermal cells denoted by white arrow), amphid, pre-vulva, posterior intestine and neurons near the rectum. Scale bar = 20 μm.

Figure S6. Knock-down of *pals-17* induces the IPR and changes intestinal mitochondria morphology. A) Representative images of *pals-5p::*GFP, *myo-2p::*mCherry IPR reporter expressing animals treated with either control or *pals-17* RNAi for 24 h starting at 20 h post-L1. Control RNAi does not induce IPR reporter, and *pals-17* RNAi induces *pals-5p*::GFP IPR reporter expression in the intestine at the 24 h timepoint shown (white arrows). *myo-2p::*mCherry is part of the same transgene and is constitutively expressed in the pharynx of transgenic animals at all life stages. Images are an overlay of DIC, GFP, and mCherry fluorescence channels. Scale bar = 100 μm. **B)** Representative images of *mgIs48[ges-1p::mito-GFP*] animals 20 h post-L1 exposed to control or *pals-17* RNAi for 24 hours. The white boxes indicate an ROI in the first ring of intestinal cells, and increased magnification showing mitochondria morphology in the ROI based on mito-GFP expression is inset. Scale bar = 15 μm, ROI scale bar = 2.5 μm. **C)** *pals-17* RNAi induces changes in intestinal mitochondria morphology, as measured by form factor. * $p < 0.05$, unpaired t-test. n = 30 ROIs analyzed per condition, two ROIs per animal, across three experimental replicates. The graph indicates mean values and error bars represent standard deviations. Different symbol shapes represent ROIs from animals imaged on different days.

Figure S1

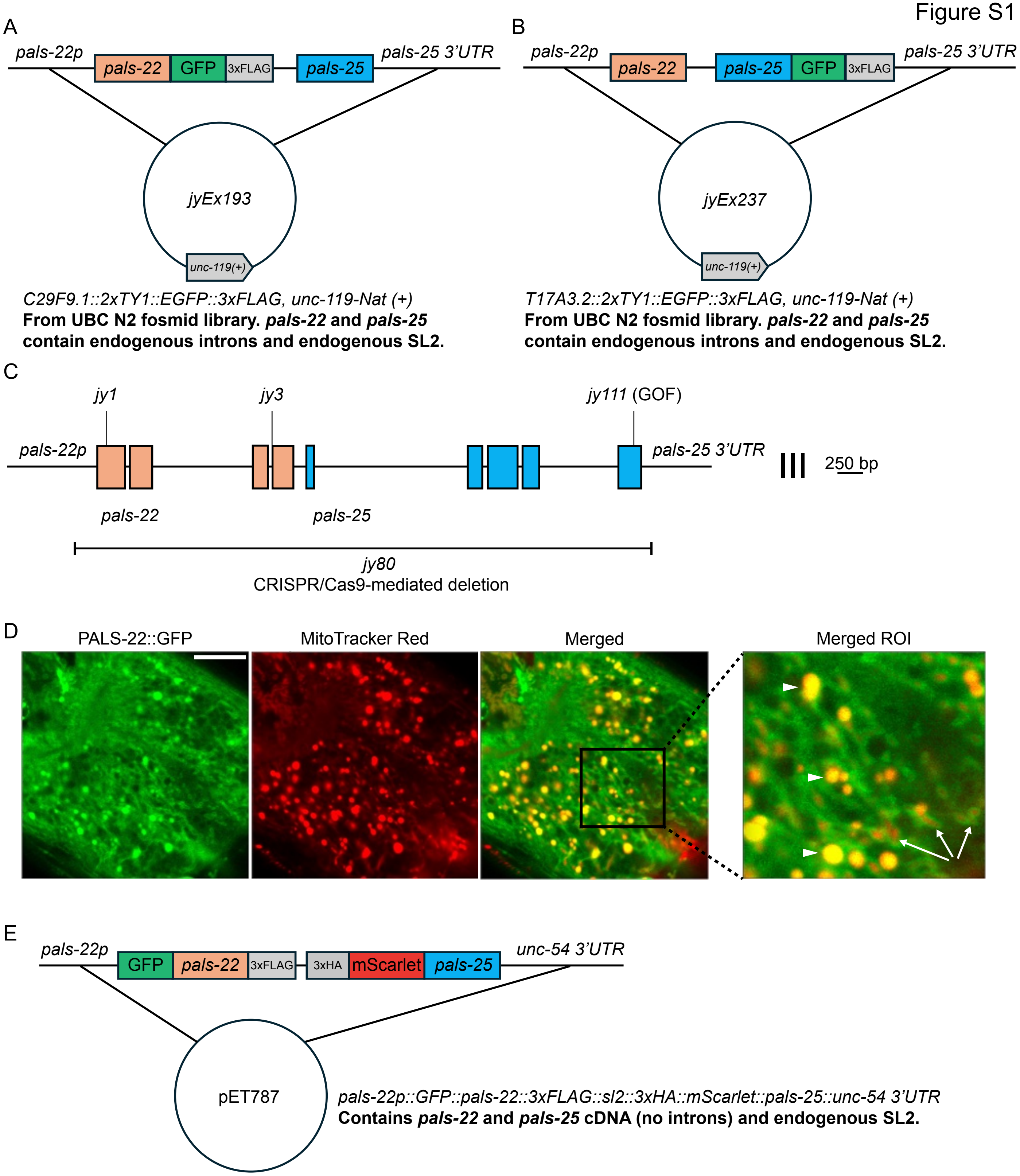
A
pals-22p
pals-22
GFP
3xFLAG
pals-25
pals-25 3'UTR
jyEx193
unc-119(+)
C29F9.1::2xTY1::EGFP::3xFLAG, unc-119-Nat (+)
From UBC N2 fosmid library. pals-22 and pals-25 contain endogenous introns and endogenous SL2.
B
pals-22p
pals-22
pals-25
GFP
3xFLAG
pals-25 3'UTR
jyEx237
unc-119(+)
T17A3.2::2xTY1::EGFP::3xFLAG, unc-119-Nat (+)
From UBC N2 fosmid library. pals-22 and pals-25 contain endogenous introns and endogenous SL2.
C
jy1
jy3
jy111 (GOF)
pals-22p
pals-25 3'UTR
250 bp
pals-22
pals-25
jy80
CRISPR/Cas9-mediated deletion
D
PALS-22::GFP
MitoTracker Red
Merged
Merged ROI
E
pals-22p
GFP
pals-22
3xFLAG
3xHA
mScarlet
pals-25
unc-54 3'UTR
pET787
pals-22p::GFP::pals-22::3xFLAG::sl2::3xHA::mScarlet::pals-25::unc-54 3'UTR
Contains pals-22 and pals-25 cDNA (no introns) and endogenous SL2.

Figure S2

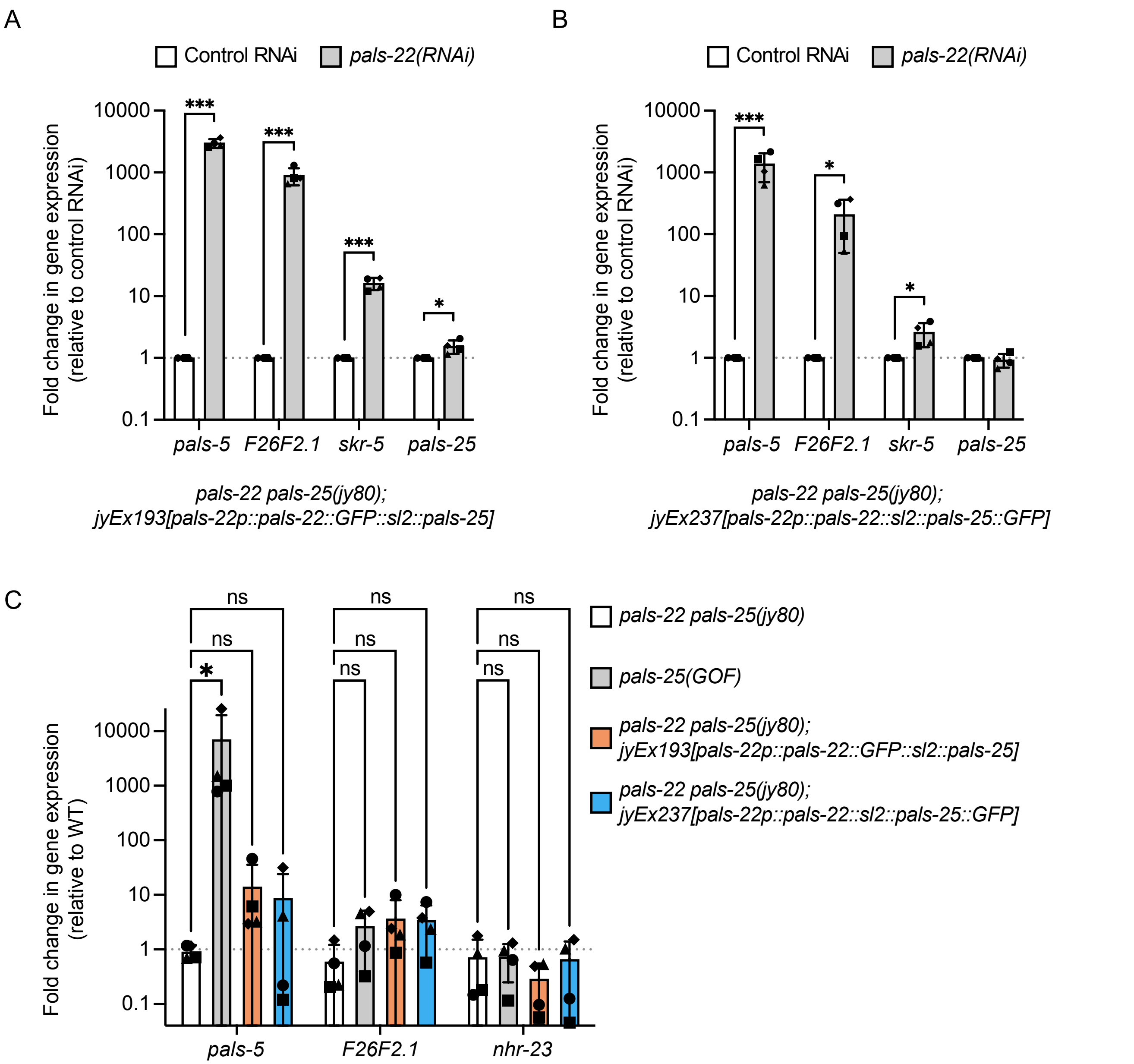
A
Control RNAi
pals-22(RNAi)
Fold change in gene expression (relative to control RNAi)
10000
1000
100
10
1
0.1
***
***
***
*
pals-5
F26F2.1
skr-5
pals-25
pals-22 pals-25(jy80);
jyEx193[pals-22p::pals-22::GFP::sl2::pals-25]
B
Control RNAi
pals-22(RNAi)
Fold change in gene expression (relative to control RNAi)
***
*
*
pals-5
F26F2.1
skr-5
pals-25
pals-22 pals-25(jy80);
jyEx237[pals-22p::pals-22::sl2::pals-25::GFP]
C
ns
*
Fold change in gene expression (relative to WT)
pals-5
F26F2.1
nhr-23
pals-22 pals-25(jy80)
pals-25(GOF)
pals-22 pals-25(jy80);
jyEx193[pals-22p::pals-22::GFP::sl2::pals-25]
pals-22 pals-25(jy80);
jyEx237[pals-22p::pals-22::sl2::pals-25::GFP]

Figure S3

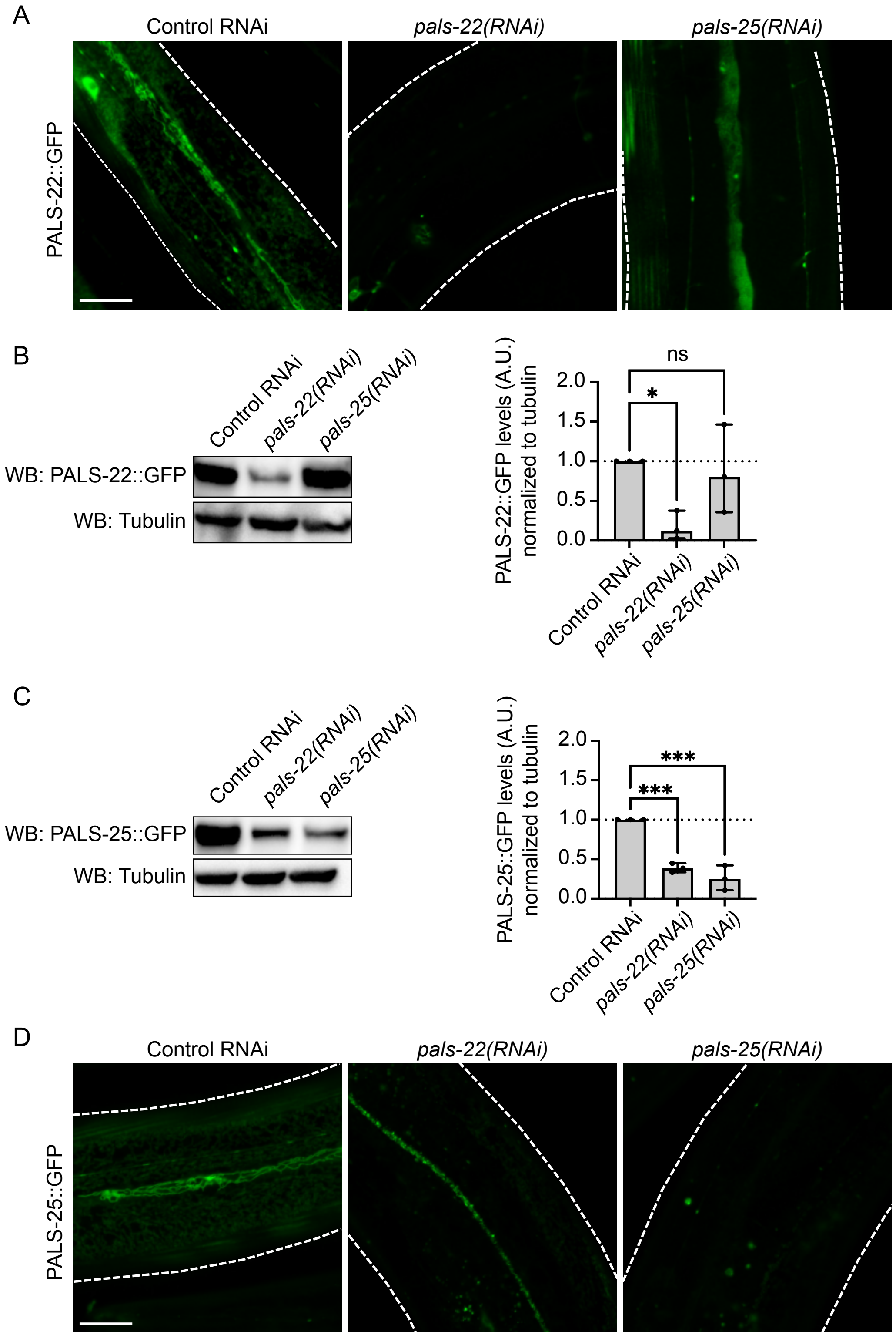

A
Control RNAi
pals-22(RNAi)
pals-25(RNAi)
PALS-22::GFP
B
Control RNAi
pals-22(RNAi)
pals-25(RNAi)
WB: PALS-22::GFP
WB: Tubulin
PALS-22::GFP levels (A.U.) normalized to tubulin
2.0
1.5
1.0
0.5
0.0
ns
*
Control RNAi
pals-22(RNAi)
pals-25(RNAi)
C
Control RNAi
pals-22(RNAi)
pals-25(RNAi)
WB: PALS-25::GFP
WB: Tubulin
PALS-25::GFP levels (A.U.) normalized to tubulin
2.0
1.5
1.0
0.5
0.0
***
***
Control RNAi
pals-22(RNAi)
pals-25(RNAi)
D
Control RNAi
pals-22(RNAi)
pals-25(RNAi)
PALS-25::GFP

Figure S4

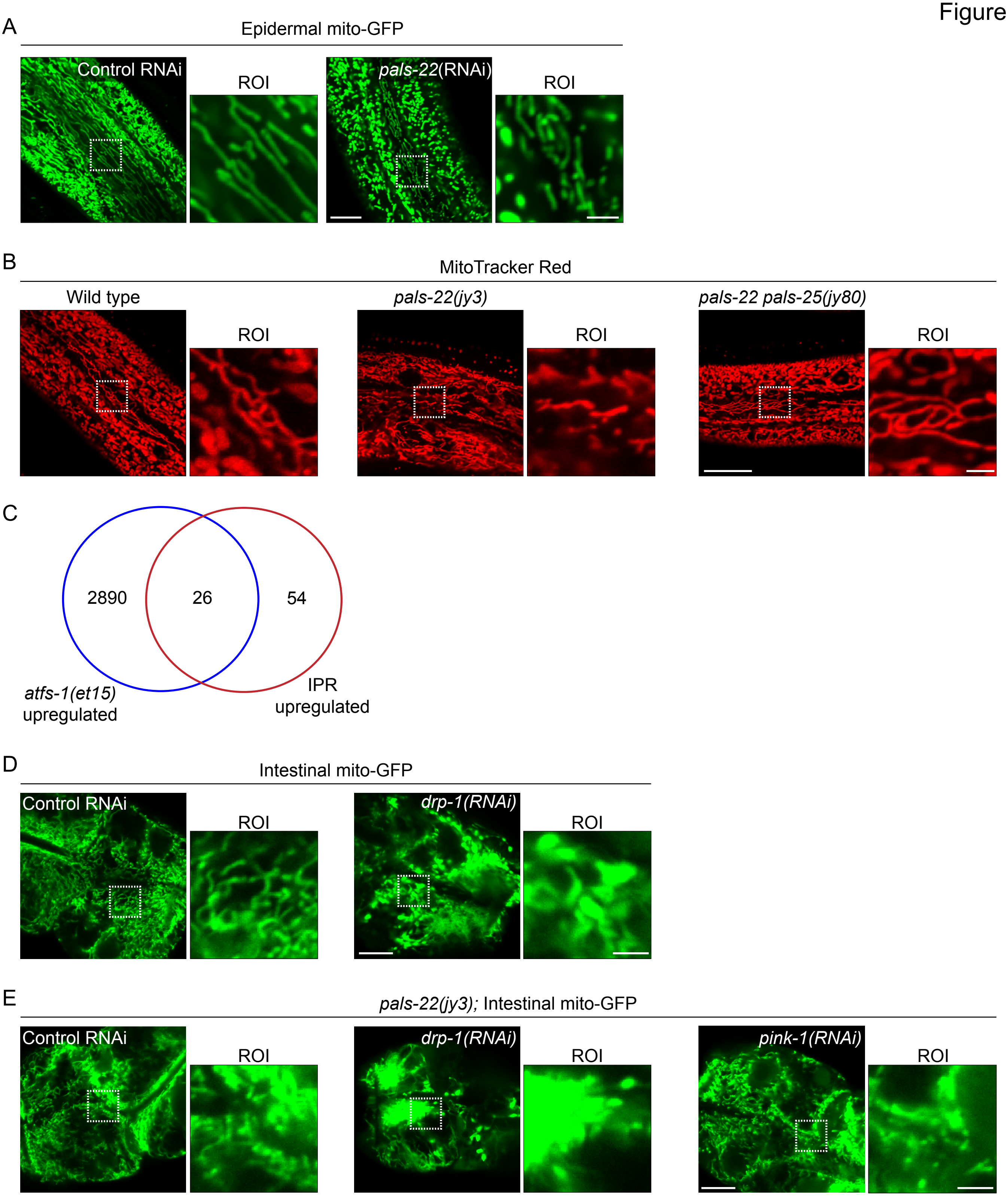
A
Epidermal mito-GFP
Control RNAi
ROI
pals-22(RNAi)
ROI
B
MitoTracker Red
Wild type
ROI
pals-22(jy3)
ROI
pals-22 pals-25(jy80)
ROI
C
2890
26
54
atfs-1(et15)
upregulated
IPR
upregulated
D
Intestinal mito-GFP
Control RNAi
ROI
drp-1(RNAi)
ROI
E
pals-22(jy3); Intestinal mito-GFP
Control RNAi
ROI
drp-1(RNAi)
ROI
pink-1(RNAi)
ROI

Figure S5

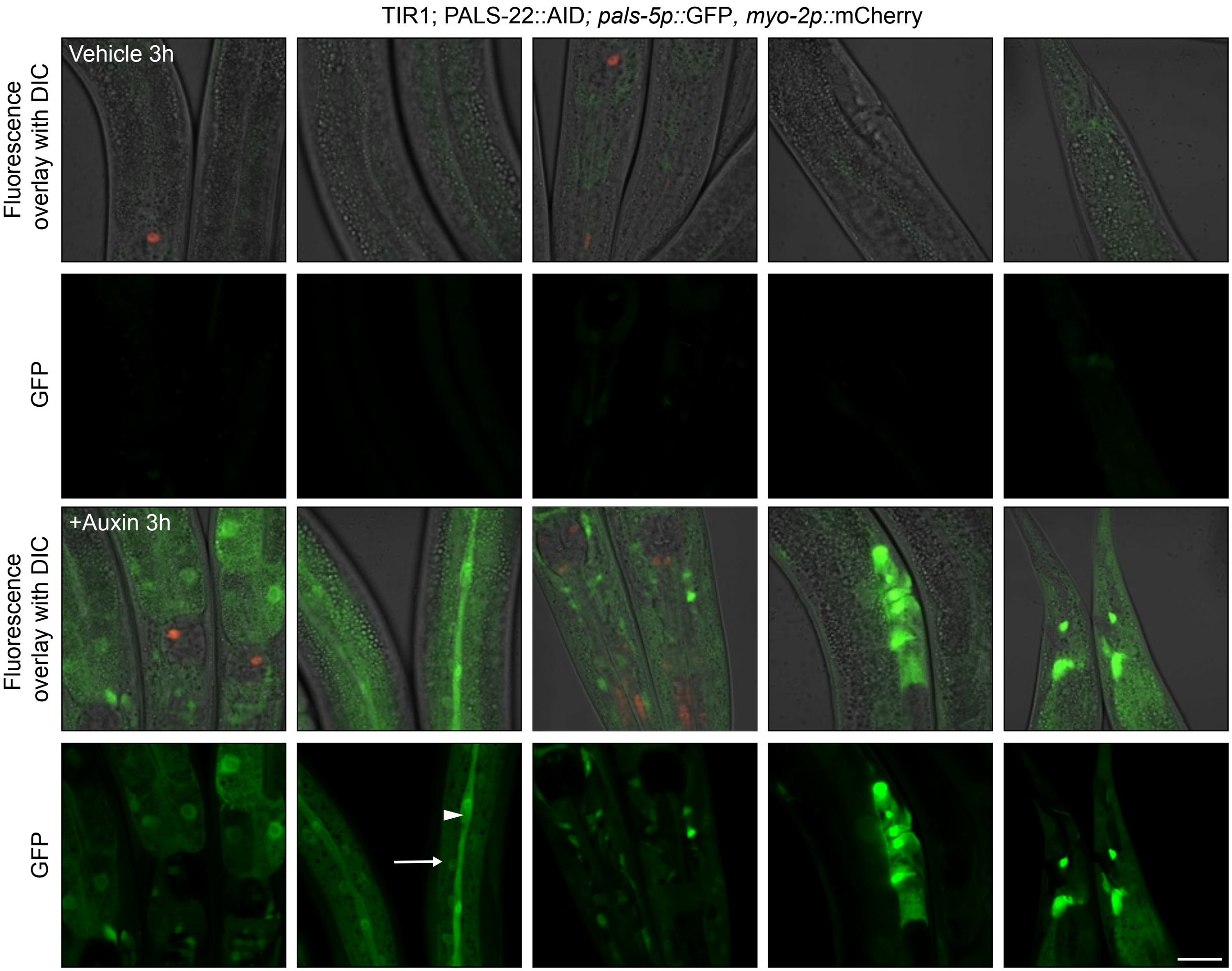
TIR1; PALS-22::AID; *pals-5p::*GFP, *myo-2p::*mCherry
Vehicle 3h
+Auxin 3h
Fluorescence overlay with DIC
GFP
Fluorescence overlay with DIC
GFP

Figure S6

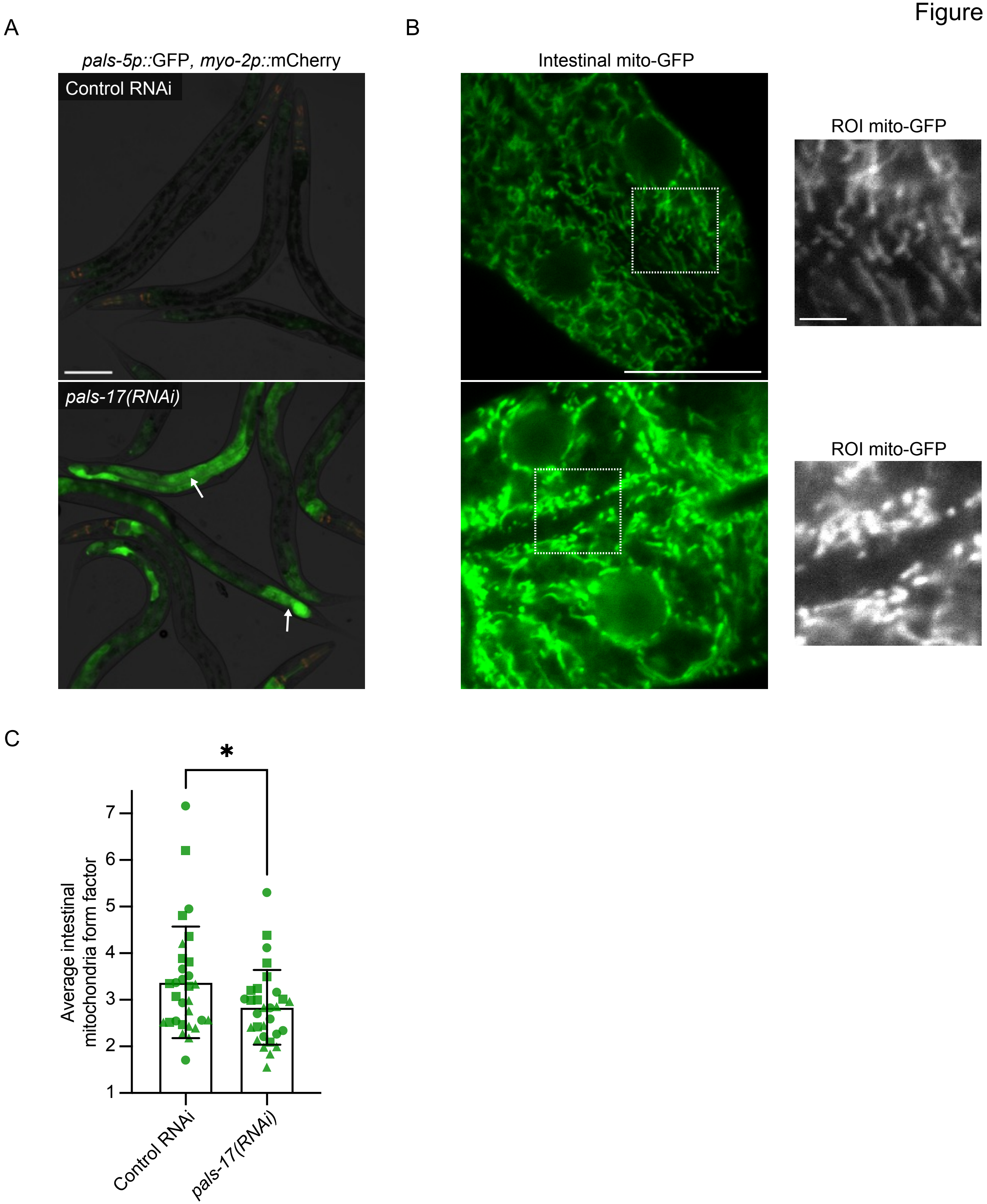
A
pals-5p::GFP, myo-2p::mCherry
Control RNAi
pals-17(RNAi)
B
Intestinal mito-GFP
ROI mito-GFP
ROI mito-GFP
C
*
Average intestinal mitochondria form factor
1
2
3
4
5
6
7
Control RNAi
pals-17(RNAi)
